# Dasatinib-Induced Pulmonary Arterial Hypertension Treated with Upfront Combination Therapy

**DOI:** 10.1155/2018/3895197

**Published:** 2018-05-20

**Authors:** Makoto Nishimori, Tomoyuki Honjo, Kenji Kaihotsu, Naohiko Sone, Sachiko Yoshikawa, Junichi Imanishi, Kazuhiko Nakayama, Noriaki Emoto, Masanori Iwahashi

**Affiliations:** ^1^Division of Cardiology, Department of Internal Medicine, Shinko Hospital, Kobe, Japan; ^2^Division of Cardiovascular Medicine, Department of Internal Medicine, Kobe University Graduate School of Medicine, Kobe, Japan; ^3^Department of Clinical Pharmacy, Kobe Pharmaceutical University, Kobe, Japan

## Abstract

Pulmonary arterial hypertension (PAH) is a rare complication of dasatinib that was approved as a first-line therapy for chronic myelocytic leukemia (CML). A 24-year-old man presenting dyspnea at rest and leg edema was admitted to our hospital. He had been diagnosed with CML and prescribed dasatinib for 4 years. Chest X-ray showed significant bilateral pleural effusion and heart enlargement. Echocardiography revealed interventricular septal compression and elevated peak tricuspid regurgitation pressure gradient of 66.7 mmHg indicating severe pulmonary hypertension. After the other specific diseases to provoke PAH were excluded, he was diagnosed with dasatinib-induced PAH. Despite discontinuation of dasatinib and intravenous administration of diuretic for two weeks, World Health Organization (WHO) functional class was still II and mean pulmonary arterial pressure (PAP) was high at 37 mmHg. Therefore, we administered sildenafil and bosentan together as an upfront combination therapy three weeks after dasatinib discontinuation. Six months later, his symptoms improved to WHO functional class I and mean PAP was decreased to 31 mmHg. Although PAH is a rare complication of dasatinib, symptomatic patients prescribed with dasatinib should have an echocardiogram for PAH screening. Moreover, the upfront combination therapy would be a useful option for symptomatic patients after discontinuation of dasatinib.

## 1. Introduction

Pulmonary hypertension (PH) is defined as an elevation in mean pulmonary arterial pressure (PAP) of 25 mmHg or more at rest. Pulmonary arterial hypertension (PAH) is a subgroup of PH and characterized by remodeling of pulmonary vasculature, resulting in increased pulmonary vascular resistance and PAP. It is reported that some drugs including dasatinib cause PAH [1].

Dasatinib, a second generated tyrosine kinase inhibitor (TKI), is widely used as a first-line therapy for chronic myelocytic leukemia (CML). Among the complications of dasatinib, PAH is a rare but severe complication. To treat dasatinib-induced PAH, some cases have demonstrated the effectiveness of pulmonary vasodilators [2]. However, data regarding the use of vasodilators are limited. Here, we present a case of dasatinib-induced PAH treated with an upfront combination therapy with vasodilators.

## 2. Case Presentation

A 24-year-old man with no past history of cardiopulmonary disease visited our hospital because of worsening of dyspnea at rest and leg edema. He had been diagnosed with CML seven years ago and initially treated with imatinib. Three years later, imatinib was switched to dasatinib (Sprycel®, Bristol-Myers; 100 mg/day) because of the incomplete molecular remission.

**Figure 1:**
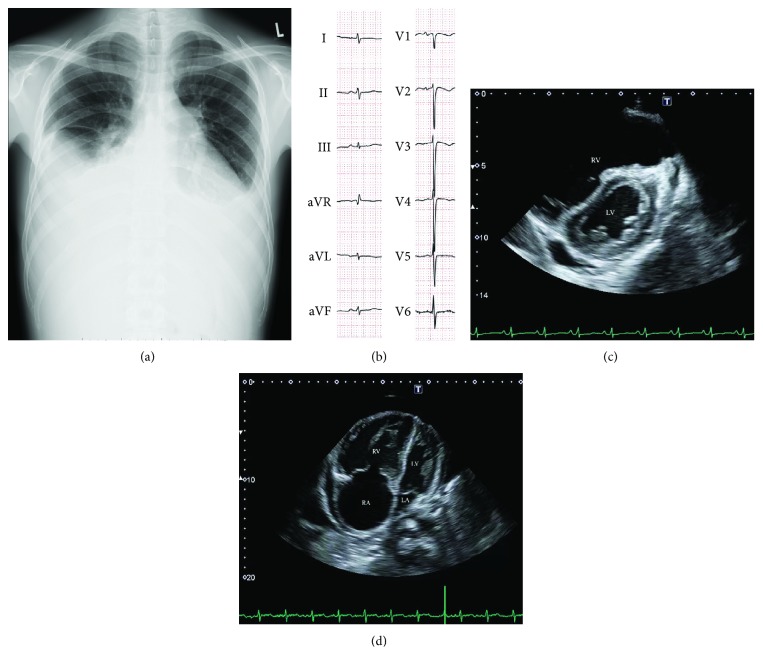
Clinical images on admission leading to diagnosis of pulmonary artery hypertension. (a) Chest X-ray on admission showed cardiac enlargement and severe bilateral plural effusion. (b) ECG suggested mild right ventricular overload and clockwise rotation. (c) Short axis view of the TTE showed flattening of the interventricular septum. (d) Apical four-chamber view of the TTE showed decreased left ventricular chamber size pressured by enlarged right ventricle. TTE: transthoracic echocardiography; LV: left ventricular; LA: left atrium; RV: right ventricular; RA: right atrium.

On admission, he developed increased nocturnal dyspnea, leg edema, and nausea. His blood pressure was 104/71 mmHg, heart rate was regular at 103 bpm, body temperature was 37.0°C, and oxygen saturation was 91% in room air. An X-ray examination found significant bilateral pleural effusion and heart enlargement (Figure 1(a)). An electrocardiogram showed low voltage in the limb lead, left axis deviation, clockwise rotation, and negative T waves in the V1-V2 lead indicating pleural effusion and right ventricular enlargement (Figure 1(b)). A transthoracic echocardiography revealed an enlarged right ventricle (D1 59 mm, D2 48 mm) with flattening of the interventricular septum during systolic and diastolic phases and severe tricuspid regurgitation with the elevated tricuspid regurgitation pressure gradient (TRPG) of 66 mmHg, indicating increased PAP and volume overload (Figures 1(c) and 1(d)). A chest contrast-enhanced computed tomography and a ventilation-perfusion lung scintigraphy showed no findings suggestive of thromboembolic disease. After the other specific diseases known to provoke PAH including human immunodeficiency virus infection, connective tissue disease, right-to-left shunt, and left heart disease were excluded, he was diagnosed with dasatinib-induced PAH in August 6, 2015.

After the admission as day 1, dasatinib was discontinued on day 2 and switched to bosutinib on day 14 which is another TKI used as a third choice for CML. In addition, bolus injection of loop diuretic (furosemide 20 mg daily from days 2 to 5) and oral diuretic (azosemide 60 mg qd from day 3) was started.

**Figure 2:**
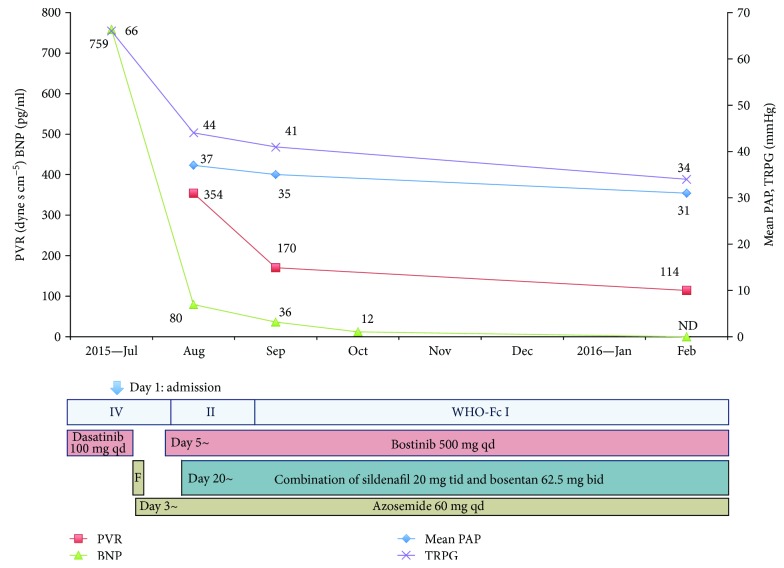
Clinical course of the case. BNP: B-type natriuretic peptide; PAP: pulmonary artery pressure; PVR: pulmonary vascular resistance; TRPG: tricuspid regurgitation peak gradient; WHO-Fc: World Health Organization functional class; F: furosemide 20 mg/day i.v. for three days; ND: not detected.

Two weeks after admission, his body weight decreased from 70.3 kg to 59.6 kg and pulmonary effusion disappeared. However, World Health Organization (WHO) functional class was still class II, and flattening of the interventricular septum and elevated TRPG (44 mmHg) was observed by the echocardiography. In right heart catheterization (RHC) on day 15, PAP was 55 (systolic)/26 (diastolic)/37 (mean) mmHg, pulmonary capillary wedge pressure was 8 mmHg, cardiac output was 6.55 l/min, cardiac index was 3.72 l/min/m^2^, and pulmonary vascular resistance was 353 dyne s cm^−5^, respectively (Figure 2).

**Figure 3:**
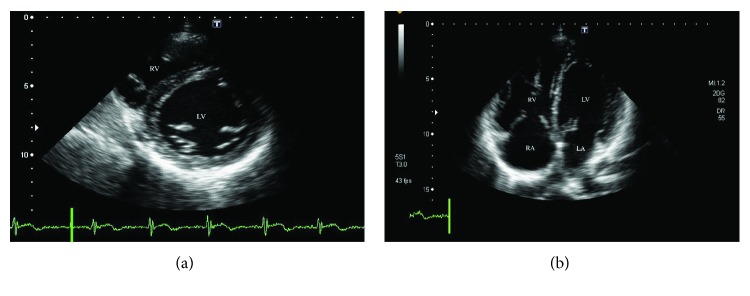
Follow-up echocardiography, 6 months after the upfront combination therapy. (a) Short axis view of the TTE showed no compression of the interventricular septum. (b) Apical four chambers view the TTE showed improvement of the right ventricular size. TTE: transthoracic echocardiography; LV: left ventricular; LA: left atrium; RV: right ventricular; RA: right atrium.

Since discontinuation of dasatinib and diuretic administration had an insufficient therapeutic effect to decrease dyspnea and PAP, we started the upfront combination therapy with sildenafil, a phosphodiesterase-5 inhibitor, at 20 mg tid, and bosentan, an endothelin receptor antagonist, at 62.5 mg bid on day 20. One month later, his symptom was improved to WHO functional class I, mean PAP measured by RHC on day 15 was slightly reduced to 35 mmHg, and B-type natriuretic peptide level was decreased from 785 to 36 pg/ml. Six months later, mean PAP measured by RHC was decreased to 31 mmHg (Figure 2). The echocardiography also showed improvements in the right ventricular diameter (D1 48 mm, D2 40 mm) as shown in Figure 3. Finally, 12 months after the upfront combination therapy and dasatinib discontinuation, TRPG evaluated by the echocardiography was decreased to 24 mmHg.

## 3. Discussion

We encountered a case of severe dasatinib-induced PAH that was improved by the upfront combination therapy of vasodilators using a phosphodiesterase-5 inhibitor and an endothelin receptor antagonist.

Dasatinib is a second generated TKI that was recently approved as a first-line therapy for CML. Dasatinib inhibits not only the BCR-ABL oncogenic tyrosine kinase in Philadelphia chromosome-positive CML but also other tyrosine kinases. Bosutinib is also approved for salvage therapy in CML. Unlike other TKIs, bosutinib does not significantly inhibit Kit protein or platelet-derived growth factor receptor [3]. Among these tyrosine kinases, Src tyrosine kinase has been identified as likely the cause of PAH. The Src tyrosine kinase is abundantly expressed in vascular tissue. Activation of Src appears to play a critical role in proliferation of smooth muscle cells and vasoconstriction. Recently, Guignabert et al. demonstrated that chronic administration of dasatinib can cause pulmonary endothelial damage and PH in rats [4]. When monocrotaline and chronic hypoxic stress were given to the rats that were treated with dasatinib, imatinib, or a control vehicle, PH and pulmonary artery wall thickening occurred in only the dasatinib-treated rats in a dose-dependent manner. They highlighted that Src inhibitory profile of dasatinib exacerbated pulmonary hypertension, which was not observed in imatinib treatment. In addition, clinical report showed that imatinib improved exercise capacity and hemodynamics in advanced PAH patients [5]. Thus, it is important to know the character and kinase pathway when we choose TKIs for CML treatment. In this case, we changed dasatinib to bosutinib as a third-line therapy because there was no report of bosutinib-induced PAH at the time of the medication switch. However, recently, some case reports showed bosutinib occasionally provoked PAH [6], which has similar inhibitory profile on tyrosine kinases as dasatinib [3]. Given the patient's refractoriness to imatinib, an alternative TKI was used, which fortunately to date has not produced recurrent PH symptoms in the patient even though there are reported cases of bosutinib-associated PH and though the putative causative pathway may be invoked by both drugs.

A French group reported the incidence of dasatinib-induced PAH as 0.45% (13 out of 2900 patients) among symptomatic patients [1]. However, according to another report, increased right ventricular pressure was observed in 5 out of 38 patients (13.2%) assessed by echocardiography [7]. This incidence was higher than that in the previous report [1], suggesting the presence of asymptomatic dasatinib-induced PAH. Since pleural effusion and systemic edema are common side effects of dasatinib, it is quite difficult to distinguish PAH from common side effects by only symptoms. Therefore, early screening by echocardiography to detect the development of PAH is necessary in symptomatic patients.

It is reported that 34 out of 36 patients (94%) with dasatinib-induced PAH recovered by discontinuation of dasatinib, but 15 out of 36 patients (41.6%) did not show complete hemodynamic recovery [8]. Moreover, in another report on dasatinib-induced PAH, mean PAP did not decrease to less than 25 mmHg even with drug discontinuation or single vasodilator therapy [1]. Based on these findings, only drug discontinuation might not enough for complete remission of PH, suggesting the relevance of aggressive PH specific therapy. In fact, in our case, with the upfront combination therapy for 12 months, TRPG evaluated by the echocardiography was decreased to 24 mmHg.

The optimal timing and combination of the vasodilator intervention are still under debate. Since SUPER-2 trial showed that 12 weeks delay of the vasodilator made the prognosis of the patients worse [9], we decided to treat the patient with the upfront combination therapy after 4 weeks of dasatinib discontinuation aiming rapid and better outcome. Recently, AMBITION trial showed that initial combination therapy with ambrisentan and tadalafil resulted in a significantly lower risk of clinical failure events for the patients with PAH [10]. In this case, we chose bosentan and sildenafil because the AMBITION trial had not been published yet at the time of medication switch. Although the combination therapy with bosentan and sildenafil does not have evidence, initial use of these two drugs efficiently ameliorated the patient's symptoms. It could be another option for dasatinib-induced PAH treatment. Further accumulation of the cases is needed.

## 4. Conclusion

We experienced a case of dasatinib-induced PAH during the treatment of CML. We should consider the upfront combination therapy for symptomatic patients with PAH after discontinuation of dasatinib. Moreover, we strongly recommend routine screening of PAH by echocardiography during dasatinib or other TKI therapy in patients with edema or plural effusion.
